# The Ins and Outs of an Online Bipolar Education Program: A Study of Program Attrition

**DOI:** 10.2196/jmir.1450

**Published:** 2010-12-19

**Authors:** Jennifer Nicholas, Judith Proudfoot, Gordon Parker, Inika Gillis, Rowan Burckhardt, Vijaya Manicavasagar, Meg Smith

**Affiliations:** ^2^School of Social SciencesUniversity of Western SydneySydneyAustralia; ^1^School of PsychiatryUniversity of New South Wales and Black Dog InstituteSydneyAustralia

**Keywords:** Non-adherence, Nonadherence, attrition, eHealth, online psycho-education program, bipolar disorder, Internet intervention

## Abstract

**Background:**

The science of eHealth interventions is rapidly evolving. However, despite positive outcomes, evaluations of eHealth applications have thus far failed to explain the high attrition rates that are associated with some eHealth programs. Patient adherence remains an issue, and the science of attrition is still in its infancy. To our knowledge, there has been no in-depth qualitative study aimed at identifying the reasons for nonadherence to—and attrition from— online interventions.

**Objective:**

This paper explores the predictors of attrition and participant-reported reasons for nonadherence to an online psycho-education program for people newly diagnosed with a bipolar disorder.

**Methods:**

As part of an ongoing randomized controlled trial (RCT) evaluating an online psycho-education program for people newly diagnosed with a bipolar disorder, we undertook an in-depth qualitative study to identify participants’ reasons for nonadherence to, and attrition from, the online intervention as well as a quantitative study investigating predictors of attrition. Within the RCT, 370 participants were randomly allocated to 1 of 2 active interventions or an attention control condition. Descriptive analyses and chi-square tests were used to explore the completion rates of 358 participants, and standard regression analysis was used to identify predictors of attrition. The data from interviews with a subsample of 39 participants who did not complete the online program were analyzed using “thematic analysis” to identify patterns in reported reasons for attrition.

**Results:**

Overall, 26.5% of the sample did not complete their assigned intervention. Standard multiple regression analysis revealed that young age (*P*= .004), male gender *(P*= .001), and clinical recruitment setting (*P*= .001) were significant predictors of attrition (F_7,330_= 8.08, *P*< .001). Thematic analysis of interview data from the noncompleter subsample revealed that difficulties associated with the acute phases of bipolar disorder, not wanting to think about one’s illness, and program factors such as the information being too general and not personally tailored were the major reasons for nonadherence.

**Conclusions:**

The dropout rate was equivalent to other Internet interventions and to face-to-face therapy. Findings from our qualitative study provide participant-reported reasons for discontinuing the online intervention, which, in conjunction with the quantitative investigations about predictors, add to understanding about Internet interventions. However, further research is needed to determine whether there are systematic differences between those who complete and those who do not complete eHealth interventions. Ultimately, this may lead to the identification of population subgroups that most benefit from eHealth interventions and to informing the development of strategies to improve adherence.

**Trial Registration:**

ACTRN12608000411347; http://www.anzctr.org.au/ACTRN12608000411347.aspx (Archived by WebCite at http://www.webcitation.org/5uX4uYwVN)

## Introduction

The science of eHealth interventions is evolving [1,2]. Drawing on quality standards for the field [3], new programs and platforms have been developed, clinical trials utilizing gold standard methodologies have been conducted, and clinical efficacy and cost effectiveness have been demonstrated [4,5]. However, participant adherence to the interventions remains an issue. A recent systematic review of 19 Internet-based psychological treatment programs found that attrition during treatment ranged from 2% to 83% with a median of 19% and a weighted average of 31% [6]. High eHealth attrition rates may be a natural and typical feature [7], but the reasons are not well known, and the phenomenon creates methodological challenges in studies evaluating eHealth applications. In this particular area, the science of eHealth interventions is still in its infancy.

Dropout rates from online programs do not differ greatly from psychotherapy delivered face-to-face. A mean rate of premature termination of 46.86% (SD 22.25) was found in a meta-analysis of 125 studies of face-to-face therapy [8], while more recent studies of attrition in face-to-face psychotherapy have reported rates of 24% in a clinical psychology service setting [9] and 33% in primary care settings [10]. However, the vast methodological differences between traditional and eHealth interventions necessitate the investigation of attrition from online interventions in their own right. Appropriate frameworks and models of eHealth attrition are needed to fully understand and assess the reasons behind dropout in order to maximize the impact of interventions. Eysenbach [7] has posited that, among other factors, “losing interest” is one common factor to both dropout to follow-up and nonusage of the application, but this has yet to be tested. Other hypothesized reasons for attrition include characteristics of the intervention such as its ease of use [7], clarity of expectations [7], and adjunctive personal contact, which can influence usage [7, 11]. User characteristics such as education level, severity of the mental health problem, need for anonymity, availability of alternative resources, and preference for treatment modalities have also been suggested [12].

Until recently, very little research has investigated nonadherence to and attrition from Internet interventions [7, 13]. A recent review of Internet interventions for anxiety and depression by Christensen et al [14] noted that many studies failed to report adherence to the content of the intervention, detailing only dropout from trial assessments. Even fewer studies have assessed the predictors of adherence, while only one [15] has formally examined participant-reported reasons for nonadherence and dropout. Although an important addition to the field, the data from that study were collected by questionnaire, which by necessity imposes restrictions on their possible richness and depth.

The paucity of research into attrition represents a significant gap in the science of eHealth interventions [7]. The necessity for such studies is emphasized by the tendency to base judgments of the utility of interventions on dropout rates. However, Proudfoot et al’s [16] survey of noncompleters of their “Beating the Blues” computer-based cognitive behavioural therapy (CCBT) for depression and anxiety found that only half the noncompleters (53%) cited negative reasons for abandoning the program. Therefore, the assertion that nonadherence and dropout are negative reflections on interventions may not always be correct in eHealth, and more investigation is required to better understand the intricacies of attrition.

As eHealth interventions have the potential to significantly enhance access to high quality cost-effective care throughout the world, reasons behind participant nonadherence, and an examination of who continues with the intervention and why is of scientific interest and crucial for the future utility of online mental health delivery.

The current study was designed to investigate patterns of adherence to an online psycho-education program for people with bipolar disorder. Bipolar disorder is a chronic illness that is characterized by periods of mania/hypomania and depression. It was earlier reported that 1.3% of the population will suffer from bipolar disorder across their lifetime; however, more recent evidence suggests a lifetime risk of 5% [17,18]. Bipolar disorder has been ranked the sixth leading cause of disability in the world, with approximately 40% of people with bipolar disorder relapsing in the first year, 60% over two years, and 75% over three years [19]. Moreover, bipolar disorder bears the highest suicide rate of all psychiatric disorders, with approximately 25% of patients attempting suicide, and 10% to 20% completing suicide [20]. Psycho-education has been shown to increase patients’ and their supporters’ knowledge of the disorder and of treatment options, improve treatment adherence, and decrease relapses and hospitalizations [21].

Our study aimed to identify participant, program, and setting factors related to nonadherence in an online psycho-education program and to fill a gap in the literature by undertaking in-depth qualitative interviews with a cross section of non-completers to understand their reasons for discontinuation. Based on previous research [13-15] we predicted that gender, age, and illness severity would influence program adherence.

## Methods

### Participants

This study is part of an ongoing randomized clinical trial (RCT) aimed at evaluating the effectiveness of an online psycho-education program in helping people newly diagnosed with bipolar disorder to adjust to their diagnosis and to gain control of their illness. Details of the randomized controlled trial have been outlined in Proudfoot et al [22]. Participants were recruited through the Black Dog Institute Mood Disorders clinic, the Black Dog Institute website [23], community mental health organizations, general practitioners and psychiatrists, and the print media. Information and flyers were placed in the clinic and on the website and were distributed to community organizations, general practitioners and psychiatrists, and their professional and support organizations. In addition, brief text advertisements were placed in 3 newspapers, 1 Australia-wide and 2 Sydney-based (see Multimedia Appendix 1). To be eligible for the study, participants had to be 18 or more years of age, had to have been diagnosed with bipolar disorder by a general practitioner or psychiatrist within the past 12 months, had to be currently seeing a health professional for the treatment of their bipolar disorder, had to be without suicidal ideation, had to have access to the Internet, had to be computer literate, had to be living in Australia, had to be able to read and write English, and had to be prepared to take part in the 6-month study. To confirm their diagnosis of bipolar disorder, participants completed the Mood Swings Questionnaire [24], and those who scored at or above the cutoff of 22 were invited to take part in the study. Power calculations based on the outcome measures showed that to detect an effect size of 0.5 between the online psycho-education program and the control group, and 0.4 between the two online programs, with a power of 80% (alpha = .05), a sample of 100 participants per group was required. To allow for attrition, we set a sample size of 140 in each of the 3 groups (ie, a sample size of 420). From January 2007 to August 2009, 370 participants were enrolled in the program and their pattern of adherence was studied as a substudy within the RCT. The results of the RCT will be reported separately when the full sample has been recruited and follow-up data have been collected.

### Interventions

Using a computer-generated randomization list, an independent researcher randomly allocated consenting participants to 1 of 3 conditions: 2 active interventions and 1 attention control condition. Those allocated to the 2 active intervention groups received an online psycho-education program for bipolar disorder either alone (Bipolar Education Program [BEP], see below) or with email support from informed supporters (BEP + IS). The allocation sequence was concealed from the researcher (author JN) who enrolled and assessed participants.

Informed supporters were expert patients with bipolar disorder who were effectively managing their condition and trained to provide email support to participants under the supervision of the research team. Informed supporters were evaluated for suitability for the study on advice from their managing psychiatrist and the judgement of study chief investigator (author JP) of their performance during the training program. Informed supporters attended an 8-hour manual-based training course developed specifically for the program, which was administered over 2 sessions by the study team. Session 1 provided an overview of the research study, including study protocol and ethical requirements and the psycho-education program. Session 2 concentrated on the role of informed supporters and included how to offer practical advice and coping strategies, particularly on issues associated with the module content, how to write sensitive and supportive emails, and how to stay within the boundaries of the role. Informed supporters attended monthly supervision sessions with the research team and were paid on an hourly basis for the supervision sessions and for the time they spent carrying out their role. All informed supporter emails to and from participants were copied to the research team for quality control and safety checking throughout the study.

The online psycho-education program consisted of 8 modules, each with associated workbooks, delivered 1 per week. It was estimated that viewing the module content and completing the workbook would take participants 30 minutes each week. The content of modules was presented as an audio-visual lecture-style slide presentation with voice narration, and topics included the causes of bipolar disorder, medications, psychological treatments, and “stay-well plans” (see Table 1). Workbooks consisted of exercises and activities designed to help participants to apply the psycho-education material to their individual situation. Specifically, the workbook activities were designed to assist participants to develop and implement their own “stay well plan.” For example, one workbook activity focussed on helping participants to identify their triggers to a depressive or manic episode. Another required them to devise a map of their support network, deciding on whom they would allow to assume what roles if they became ill.

The attention control condition consisted of online information about bipolar disorder presented in text as bullet points, of no more than 2 pages in length. It was matched on duration (8 weeks) and structure (1 module per week) to the intervention conditions and contained a “workbook” containing a brief quiz (4 questions) relating to the content of the module and a mood chart, similar to the active conditions. All participant workbook responses and mood charts were monitored by the research team for any reports of suicidal ideation or extremes in mood. Participants reporting these states were contacted by the research team via email and advised to consult their health professional. In extreme cases, the clinical psychologist on the research team contacted the participant’s doctor.

**Table 1:** Content of the Online Bipolar Education Program

Module	Topic	Content
1	Diagnosing bipolar disorder:	The importance of detection, diagnosis, and management of the bipolar disorders and distinguishing them from other conditions such as attention deficit/hyperactivity disorder, anxiety states, personality styles, and, in particular, schizophrenia
2	The causes of bipolar disorder	Genes, neurochemistry, hormones, environmental factors, stress, and personal and family background
3	Medications for bipolar disorder	Mood stabilizers, antidepressants, antipsychotics
4	Psychological treatments	Cognitive behaviour therapy; narrative therapy; solution-focussed therapy; pinpointing “early warning signs,” the signals that an episode of depression or mania may be on the horizon
5	Stay-well plans	How to reduce stress, minimize risks and maximize the chances of staying well; identifying personal triggers to illness episodes
6	Carers and support networks	Developing a contingency plan about what to do if they become unwell; considerations: extra medication, finances, work, additional treatment(s), and who to allow to help them make those decisions
7	Lifestyle changes	The benefits of establishing routines for regular sleep times and relaxation, taking medication, exercising, eating healthy foods, drinking less alcohol and caffeine, avoiding stress
8	Person first, illness last, and conclusion	People with bipolar disorder have an illness, but they themselves are not the illness; steps for setting up and implementing an action plan to stay well with bipolar disorder

### Procedure

Participant adherence was monitored throughout the study to identify noncompleters. Adherence was defined as active use (completion and return of workbooks) and sufficient dose (completion of 4 or more sessions) of the program. Participants who returned 3 or fewer completed workbooks were considered “noncompleters.” In total, 370 participants took part in the quantitative study to identify predictors of attrition.

In addition, those who met criteria for noncompletion were contacted at the 6-month follow-up point and invited to participate in a semistructured telephone interview about their impressions of the program and their reasons for discontinuation. To encourage nonresponders to participate, the interviews were not audio taped but participants’ responses were transcribed in real time. We employed the standard qualitative sampling technique of “sampling to saturation” which rests not on generalizability nor on representativeness, but on notions of “saturation,” that is, sampling is continued until the point is reached at which no new information or insights are obtained [25]. All recruited participants had completed the intervention phase of the randomized controlled trial and, consistent with best-practice qualitative method, we continued sampling as participants reached the 6-month follow-up point until no new insights were gained from the interviews. We contacted participants prior to the actual interview to allow them to choose a time that would suit them for the interview. We also asked interviewees general questions about their health at the beginning of the interview to get an indication of their current state. Participants were sent a double cinema pass for taking part in the interview. The study was approved by Human Research Ethics Committee of the University of New South Wales.

### Measures

Participants completed baseline questionnaires before taking part in the online program. These included the Goldberg Anxiety and Depression Questionnaire [26] consisting of 2 subscales, each of 9 items rated with a yes/no response. “Yes” responses were summed, producing a possible range of scores from 0 to 9, with higher scores representing more severe anxiety/depression. Details of the age that participants experienced their first symptoms of mania and depression and their current self-rated mood state (normal, depressed, high, or mixed) were also collected, as well as a range of demographic information including age category, gender, marital status, education level, and current employment status. Age category, rather than exact age, was collected as our pilot testing showed that it was more acceptable to potential participants and, therefore, they were more likely to supply it. As participants were sent modules weekly—regardless of whether they had completed the previous week’s workbook—we measured attrition by the number of participants “forever lost” from the program and their last completed workbook, as well as the total number of workbooks completed.

A specially designed semistructured interview schedule was used to gather noncompleters’ perceptions of the online bipolar psycho-education program, their health status, and their reasons for discontinuation. The health assessment portion of the interview consisted of questions measuring perceived self-control and understanding of bipolar disorder. Participants were also asked to rate their mental health at the time of discontinuation and whether they felt that depression or mania compromised their ability to do the program. The second part of the interview assessed participants’ perceptions of the online program and reasons for discontinuation. Questions related to motivations for and expectations upon joining the study, as well as their comments about the main program elements, the module information, the workbooks, their overall thoughts of, and what they would change about, the online program as a whole, how often they accessed the information, and their reasons for discontinuation. Whether participants' expectations of the study were met and their thoughts on the online format were also explored.

### Analysis

Exploratory descriptive analyses and chi-square tests were conducted to compare the completion rates of the groups of participants. Because the majority of people with bipolar disorder are diagnosed in their twenties and an inclusion criterion for the study was that participants had to have been diagnosed within the last 12 months, we dichotomized the age categories into 18 to 29 years and over 30 years for analysis. Standard multiple linear regression was used to explore predictors of attrition; the number of workbooks completed was the studied outcome.

Noncompleter interviews were analyzed using “thematic analysis” [27] to identify patterns in reported reasons for attrition. This method entails allowing the data to inform major themes derived from the participants’ responses to the questions and issues raised at interview. It involves organizing and describing the data in rich detail within a theoretical framework. In contrast to other forms of qualitative analysis, thematic analysis is not wedded to any preexisting theoretical framework, although the theoretical position used in the analysis is made clear [27]. In our analysis, we used an essentialist or realist theoretical approach, in which participants’ experiences, meanings, and reality are examined in an inductive way, in contrast to other frameworks which focus on, for example, the manner in which participants’ meanings are “constructed’ within the broader context of society. Participants’ interviews were analyzed by two members of the research team (authors JN and RB). Recurrent themes were identified and coded, and discrepancies in theme identification were resolved by discussion. Consistent with thematic analysis procedures, themes rather than numbers were analyzed and reported.

## Results

**Figure 1:**
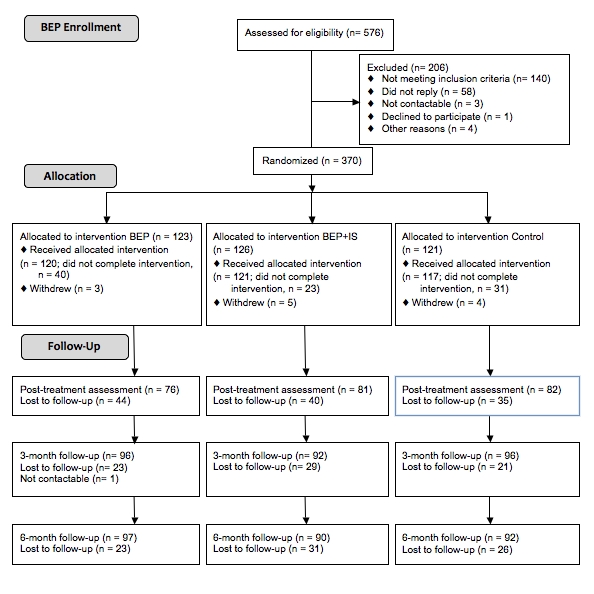
Flow diagram

Originally, 370 participants took part in this study; however, the data from 12 participants were excluded from analysis as these participants subsequently withdrew. Participant flow for the attrition substudy within the RCT is reported in Figure 1.

Of the sample of 358 participants, 69.8% (250) were female, 28.8% (103) were under 30 years of age, 45.5% (163) were married, 70.7% (253) were tertiary educated, and 57.5% (206) were in full-time employment. The mean anxiety score at baseline was 6.97 (SD 2.16), while the mean depression score at baseline was 6.47 (SD 2.11). Further details of the sample are shown in Table 2.

**Table 2:** Participant demographic characteristics

		BEP	BEP + IS	Control
		n = 120	n = 121	n = 117
		n (%)	n (%)	n (%)
**Gender**				
	Male	38 (31.7%)	32 (26.4%)	38 (32.5%)
	Female	82 (68.3%)	89 (73.6%)	79 (67.5%)
**Age**				
	18-29	40 (33.3%)	34 (28.1%)	29 (24.8%)
	30-39	43 (35.8%)	39 (32.2%)	45 (38.5%)
	40-49	21 (17.5%)	35 (28.9%)	30 (25.6%)
	50-59	12 (10.0%)	12 (9.9%)	11 (9.4%)
	60+	4 (3.3%)	1 (0.8%)	2 (1.8%)
**Marital status**				
	Never married	40 (33.3%)	41 (33.9%)	35 (29.9%)
	Married	53 (44.2%)	54 (44.6%)	56 (47.9%)
	Separated or divorced	18 (15.0%)	21 (17.4%)	21 (17.9%)
	Other	9 (7.5%)	5 (4.0%)	5 (4.3%)
**Highest education level**				
	Primary school	1 (0.8%)	0 (0%)	2 (1.7%)
	Secondary school	33 (27.5%)	35 (28.9%)	34 (29.1%)
	Tertiary education	86 (71.7%)	86 (71.1%)	81 (69.2%)
**Employment**				
	Employed (full- or part-time)	66 (55.0%)	71 (58.7%)	69 (59.0%)
	Unemployed	7 (5.8%)	7 (5.8%)	11 (9.4%)
	Full-time education	10 (8.3%)	12 (9.9%)	3 (2.6%)
	Unable to work due to sickness	16 (13.3%)	8 (6.6%)	14 (12.0%)
	Looking after home/family	11 (9.2%)	8 (6.6%)	7 (6.0%)
	Retired	2 (1.7%)	1 (0.8%)	5 (4.3%)
	Other	8 (6.6%)	14 (11.6%)	8 (6.8%)

Of the noncompleting participants, 44 were invited to be interviewed regarding their impressions of the program and their reasons for nonadherence, of whom 39 agreed. A further 26 noncompleting participants were eligible, but we were unable to contact them. Participants from all 3 study groups were interviewed, 16 from the unsupported intervention group (BEP), 9 from the supported BEP intervention group (BEP + IS), and 14 from the minimal information control group. Of these 39 noncompleting participants, 22 (56%) were female, 20 (51%) were aged less than 30 years of age, 14 (36%) were married, 29 (74%) were tertiary educated, and 24 (62%) were in full-time employment.

### Attrition Patterns

**Figure 2:**
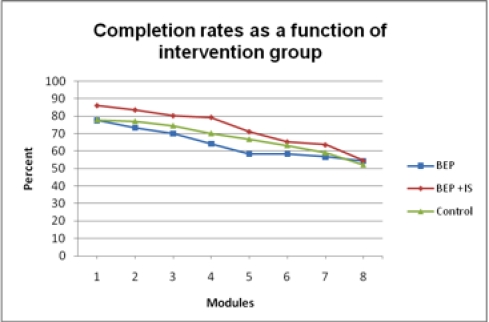
Completion rates for each of the 8 modules by intervention group: Bipolar Education Program (BEP); Bipolar Education Program with email support from informed supporters (BEP + IS); and minimal information about bipolar disorder (control)

The attrition patterns of the three 8-module interventions are presented in Figure 2.

Across the 3 interventions, there was a 73.5% (263/358) completion rate throughout the 8-week intervention, with the remaining 26.5% (95/358) of participants returning 3 or fewer module workbooks. Furthermore, 44.7% (160/358) of participants returned all 8 module workbooks, whereas 15.4% (55/358) did not return any module workbooks. Adherence was significantly higher in the supported intervention (98/121, 81.0%) compared with the unsupported (80/120, 66.7%) intervention (χ^2^_1,241_ = 6.4, *P* = .01).

### Predictors of Attrition

The results of the standard multiple regression analyses are presented in Table 3.

Significant predictors of attrition were male gender, young age, and recruitment via the Black Dog Institute clinic rather than the other recruitment avenues. Males were estimated to complete an average of 0.98 fewer workbooks than females, holding all other variables constant. Participants over 30 years of age were estimated to complete an average of 1.04 more workbooks than those under 30 years of age, and those recruited from other avenues, on average, completed 1.77 more workbooks than those recruited from the clinic, holding all other variables constant for each. Level of symptomatology, highest level of educational attainment, and baseline depression and anxiety scores did not significantly contribute to the overall model. The total variance explained by the model was 14.6% (F_7,330_ = 8.08, *P* < .001).

**Table 3:** Predictors of attrition

Variable	Coefficient	Standard Error	*P*
Gender (female vs male)	-.98	.36	.001
Symptomatic at recruitment	.24	.33	.46
Age (old vs young)	1.04	.36	.004
Anxiety preintervention score	-.02	.08	.83
Depression preintervention score	-.13	.08	.66
Method of recruitment (other vs clinic)	1.77	.34	.001
Highest level of education achieved	-.11	.34	.75

### Participant-Reported Reasons for Nonadherence

There were no statistically significant differences in baseline measures between interviewed participants and program completers or noncompleters who we were unable to contact or who declined to be interviewed.

A qualitative analysis of the interview transcripts elicited a number of key themes regarding reasons for noncompletion of the intervention. They related to participants’ health, characteristics of the online interventions, and practical issues, as detailed below. Participant ratings of their mental health on 10-point scales at the point of discontinuation yielded the following: mean reported “general mental well-being” was 6.18 (SD 2.5) (where 0 = “normal” and 10 = “worst”); mean reported “control over their bipolar disorder” was 6.94 (SD 2.18) (where 0 = “no control” and 10 = “extreme control”); and mean reported “understanding of their bipolar disorder” was 7.17 (SD 2.12) (where 0 = “no understanding” and 10 = “understand very clearly”).

### Key Themes of Qualitative Analysis

#### Discontinuation Due to the Illness Itself

Many interviewees reported that, while they were able to complete the modules and workbooks when well, being in an acute phase of the illness interfered with their ability to participate in the program. Those in a depressive phase of the illness found the lack of energy and motivation common to depression a significant hurdle to completing the program.

Female, 18-29 years, BEP group The biggest problem I have with my bipolar disorder is consistency; when I’m down I can’t even brush my teeth or get up in the morning. So doing an education program with workbooks was beyond me.

Male, 18-29 years, BEP+IS group A very short while after doing the program I fell into another episode, a depressive episode, and pretty much stopped doing everything, the program included.

Participants who experienced episodes of mania during the study discussed how they became distracted by their manic symptoms and were unable to complete the online modules.

Female, 30-39 years, Control group My highs interrupt my ability to see things through, and I get caught up in my highs.

Male, 40-49 years, BEP+IS group I often go walking when having highs because I have to keep moving, so I didn’t want to sit at a computer.

Thus, the nature of the illness itself made it difficult for some participants to continue their involvement in the program. This was the most common theme in terms of reasons for discontinuation.

#### Did Not Want to Think About Illness

Several participants reported that they found receiving weekly information about their disorder confronting or overwhelming. Many said they did not want to think about their illness and instead wanted to put it out of their minds.

Male, 40-49 years, BEP group I found it quite confronting, and reading the information made me feel uncomfortable, thinking that these issues related to me—I preferred the ostrich approach.

Female, 50-59 years, BEP+IS group [I] found it difficult to sit down and do those things. I got into an anxiety and went off to do other things. I didn’t really want to sit down and think about it.

Some participants reported that they were not ready to accept their diagnosis of bipolar disorder and so didn’t relate to the program’s information and practical advice. As the wider study was investigating the utility of the program in those newly diagnosed, some expressed the opinion that they may have enrolled in the program too soon after their diagnosis.

Male, 18-29 years, control group I wasn’t ready to accept the illness. At that stage after diagnosis I wasn’t willing to change my life according to the program.

#### The Online Program

The online bipolar psycho-education program itself was identified as a reason for discontinuation by a few of the participants who received that intervention. Most commonly, the information was regarded as too basic or simplistic, and those participants reported that they were already aware of a lot of the content before commencing the program.

Male, 18-29 years, BEP group The information in the modules was too general and too limited.

Other participants said they were dissatisfied with the program because they expected personally tailored information or feedback, which was beyond the scope of the current program.

Male, 40-49 years, BEP group I wanted something more about me specifically, as opposed to talking about general issues.

There were also comments around the layout of the programs and the amount of personal information participants were required to disclose, but these were raised by single participants and, therefore, represented a minority opinion.

#### Feeling Well

Some participants reported ceasing to utilize the program after they gained what they wanted from it or once their mood had stabilized. Other participants indicated that they worked through the modules but did not complete the associated workbooks.

Male, 50-59 years, BEP+IS group I was so self-absorbed at the time that I was only interested in the information [rather than in returning workbooks].

It was also reported by some participants that they no longer felt the need to participate in the program once their mood had stabilized and they were feeling well. This response was associated with another issue raised in the interviews, whereby a number of participants stated that they would reaccess the programs’ information after the study was completed if/when they were feeling depressed.

Female, 30-39 years, control group Things really improved for me…I just felt really good and didn’t really feel like I had that much to offer in regard to finding out more about it.

#### Time Pressures and Competing Demands

Time-related factors such as being too busy or life being too hectic were also given as reasons for discontinuation. Such reasons included being busy at work, moving house after signing up for the program, and having more important focuses.

Female, 18-29 years, control group I didn’t have the time, and with everything else, it wasn’t a priority.

However, lack of motivation was also a commonly mentioned reason, such as being forgetful or lazy about completing the program.

Female, 18-29 years, control group I have issues with procrastination. I suppose laziness is the only reason.

In these cases, the motivational problems were cited as personality characteristics, as distinct from symptoms of the bipolar disorder.

## Discussion

A comparison of adherence rates from the 3 participant groups within the large sample showed significant differences between the groups. Participants who were supported by an expert patient “informed supporter” were significantly more likely to adhere to the program compared with those who worked through the intervention modules and workbooks alone. This is consistent with previous research, which has found that guided interventions are associated with better adherence than fully automated interventions [4]. Interestingly, adherence was poorer in the unguided intervention group than in the control condition, although the difference did not reach statistical significance. Overall, our attrition rate of 26.5% is comparable to the 31% weighted average from the systematic review of 19 Internet-based psychological treatment programs [6]. It is also comparable to the attrition rate of 21% from the meta-analysis of Web-based and non-Web-based self-care interventions for chronic illness conducted by Wantland et al [28], but it is lower than the 47% dropout rate found in the older meta-analysis of psychotherapy programs delivered face-to-face [8].

Participants recruited through the Black Dog Institute Mood Disorders Clinic were significantly less likely to adhere to the program than those recruited through the other avenues. The reason for this is unclear but may have been due to clinic-recruited participants on the whole having been very recently diagnosed (often the same day as recruitment to the study) and, while they opted to take part in the study, perhaps they needed more time to come to terms with the diagnosis in order to gain more from the online psycho-education program. It is unknown whether completing the program in the clinic setting rather than at home might have enhanced adherence for this subgroup of participants. Certainly other e-mental health programs that have been delivered in clinic settings have had good rates of adherence [29].

Our finding that young age and male gender predicted nonadherence supports that of previous e-mental health findings [15] but is in contrast to research involving face-to-face treatments, such as the study by Strom et al [30], which reported that such demographic variables did not predict patient adherence across health conditions. While the relationship between gender and attrition may be mediated by other variables in the online environment [6], reaching younger males and keeping them engaged represent two distinct challenges in e-mental health research.

Similar to studies by Lange et al [15] and Strom et al [30], we did not find a significant association between education level and attrition. This is in contrast to research involving face-to-face therapies that found that clients who were from minority ethnic backgrounds, lower income groups, or were less educated were more likely to terminate therapy prematurely [8]. Although our finding does not shed any light on the commonly held belief that more highly educated users of online interventions preferentially gain greater benefit, it does point to the need for further research to tease out the relationship (if any) between benefit from online interventions and attrition.

Among people with depression, higher symptom severity has been shown to be a predictor of decreased adherence, whereas lower generalized anxiety symptom levels has predicted better adherence [14]. However, in our study, neither being symptomatic at the time of recruitment nor the severity of baseline depression or anxiety symptoms were predictors of adherence. Participant-reported reasons for nonadherence from the interviews indicated that difficulties associated with the acute phases of the illness were common reasons for nonadherence. Yet, wellness also influenced participation. The latter finding supports the hypothesis proposed by other eHealth researchers [12] that a positive factor “e-attainment” may be the root cause of some nonadherence, that is, eHealth users cease using the intervention because they feel they have achieved as much as they wish from it. This phenomenon is particular to eHealth, probably because of the relative ease with which users can disconnect, and it warrants further research.

Not wanting to think about their illness was another reason for discontinuation mentioned by participants, and this can be interpreted in a number of ways. It may be a form of denial about the diagnosis or a need to first understand some of the more existential questions associated with the diagnosis, such as what it means about the participant as a person, that is, “who am I?” Both explanations have been documented in other studies [31]. Program factors such as the information being too general and not personally tailored were the major dissatisfactions cited by some participants in the 2 intervention arms of the study.

In summary, the key reasons for nonadherence were, in the main, participant-related, and while some of the reasons given in the qualitative interviews concerned the intervention, we were unable to explore program factors to the same extent in our quantitative study. It was interesting to note that none of the reasons for attrition related to the online setting for the intervention and the study. Our findings are consistent with studies of face-to-face treatments, in which very few variables have emerged as significant predictors of premature termination from face-to-face therapy when attrition studies have been aggregated, despite the multitude of variables that have been examined in that context including client, therapist, and program-related factors. While the only consistent findings in face-to-face settings have related to socioeconomic variables [9], further research is needed to confirm whether the same set of variables exists across an aggregation of online studies.

Weaknesses of this study include the fact that workbook returns were used as the indicator of active participation and adherence. Other metrics may have been more precise, such as logs of page views or time spent on the website. Second, the sample of interviewees was not representative, as it was a nonprobabilistic sample, purposively selected. There is also a potential for recall bias in the interviews, as participants’ current mood state was not recorded at the time of interview. Additionally, the nature of the sample (people with severe mental illness) and the type of online intervention (psycho-education rather than treatment) limits the generalizability of results from the quantitative study to other online interventions for high prevalence conditions.

Nevertheless, the study highlights a number of issues surrounding attrition that are of relevance to eHealth researchers. Further research is needed to methodologically investigate nonadherence and attrition using comprehensive interviews and prediction models to assess whether any systematic differences exist between those who complete interventions and those who do not and between those who drop out early in an intervention versus those who drop out later. Ultimately this will allow the identification of population subgroups who most benefit from eHealth interventions and will inform the development of strategies to improve adherence. The study of attrition is essential for the future efficacy and utility of online interventions.

## Multimedia Appendix

Advertisement for bipolar education study

## Abbreviations

BEP: Bipolar Education Program
CCBT: computer-based cognitive behavioural therapy
IS: Informed Supporters
RCT: randomized controlled trial
