# Supplementary material for: The Ins and Outs of an Online Bipolar Education Program: A Study of Program Attrition
Source: J Med Internet Res. 2010 Dec 19;12(5):e57. doi: 10.2196/jmir.1450 (PMC3057316; doi:10.2196/jmir.1450)
Supplement: Supplementary file 1 [file jmir_v12i5e57_app1.pdf]

Multimedia Appendix 1

THE UNIVERSITY OF NEW SOUTH WALES

Education about Bipolar Disorder?

The University of New South Wales is conducting a clinical trial evaluating a **NEW** education program on Bipolar Disorder, which is designed to help people gain control of their condition. The program consists of 8 online information sessions with associated workbooks tasks.

We are seeking people for our free clinical trial who:

- Have been diagnosed with Bipolar Disorder within the last 12 months
- Are Being seen on a regular basis for treatment by a mental health professional
- Have a valid email address and access to a computer and the internet
- Can undertake 8 weekly online session of about 30mins each
- Age aged over 18

If you are interested in participating or would like further information please email: Onlineprograms@unsw.edu.au
